# Absorption, Metabolism, and Excretion by Freely Moving Rats of 3,4-DHPEA-EDA and Related Polyphenols from Olive Fruits (*Olea europaea*)

**DOI:** 10.1155/2016/9104208

**Published:** 2016-01-19

**Authors:** Shunsuke Kano, Haruna Komada, Lina Yonekura, Akihiko Sato, Hisashi Nishiwaki, Hirotoshi Tamura

**Affiliations:** ^1^The United Graduate School of Agricultural Sciences, Ehime University, 3-5-7 Tarumi, Matsuyama 790-8566, Japan; ^2^The Graduate School of Agriculture, Kagawa University, 2393 Ikenobe, Miki-cho, Kagawa 761-0795, Japan; ^3^Faculty of Agriculture, Ehime University, 3-5-7 Tarumi, Matsuyama 790-8566, Japan

## Abstract

Absorption, metabolism, and excretion of 3,4-DHPEA-EDA, oleuropein, and hydroxytyrosol isolated from olive fruits were newly evaluated after oral and intravenous administration in freely moving rats cannulated in the portal vein, jugular vein, and bile duct. Orally administered 3,4-DHPEA-EDA, an important bioactive compound in olive pomace, was readily absorbed and metabolized to hydroxytyrosol, homovanillic acid, and homovanillyl alcohol, as shown by dose-normalized 4 h area under the curve (AUC_0→4 h_/Dose) values of 27.7, 4.5, and 4.2 *μ*M·min·kg/*μ*mol, respectively, in portal plasma after oral administration. The parent compound 3,4-DHPEA-EDA was not observed in the portal plasma, urine, and bile after oral and intravenous administration. Additionally, hydroxytyrosol, homovanillic acid, and homovanillyl alcohol in the portal plasma after oral administration of hydroxytyrosol showed 51.1, 22.8, and 7.1 *μ*M·min·kg/*μ*mol AUC_0→4 h_/Dose, respectively. When oleuropein, a polar glucoside, was injected orally, oleuropein in the portal plasma showed 0.9 *μ*M·min·kg/*μ*mol AUC_0→4 h_/Dose. However, homovanillic acid was detected from oleuropein in only a small amount in the portal plasma. Moreover, the bioavailability of hydroxytyrosol and oleuropein for 4 hours was 13.1% and 0.5%, respectively. Because the amount of 3,4-DHPEA-EDA in olive fruits is about 2-3 times greater than that of hydroxytyrosol, the metabolites of 3,4-DHPEA-EDA will influence biological activities.

## 1. Introduction

Olive oil and table olives (*Olea europaea L.*) are major products in food markets all over the world and are important as dietary sources of many polyphenols such as oleuropein, oleuropein aglycones, oleocanthal, hydroxytyrosol, and luteolin, which exert biological effects such as inhibiting LDL oxidation [1–4] and atherosclerosis [5, 6], reducing postprandial blood glucose levels and the risk of diabetes [7–9], and inhibiting inflammation [10, 11]. Therefore, studies on the absorption and metabolism of olive bioactive compounds are essential to determine which compounds are biologically important in the human body after the consumption of olive products. Research works addressing the oral absorption and metabolism of olive bioactives in humans are often done by the detection of the polyphenols and related metabolites in the urine and the systemic venous blood [12–18]. Cell-based assays and the rat everted gut sac have been used to overcome the limitations of studies done in humans but would not provide data on first-pass and systemic metabolism and excretion, which require the use of a living animals. The use of small laboratory animals such as the rat is convenient, especially when measuring the absorption of compounds that are not commercially available or isolated in house, but in most cases the analysis of circulating metabolites can only be done in sacrificed animals [19–21]. Recently, the use of jugular cannulated rats enabled the study of the kinetics of absorption, metabolism, and excretion anthocyanins in freely moving rats, providing bioavailability data under more natural conditions for the animal [22]. However, to get a more accurate evaluation of absorption, the portal vein cannulation has the great advantage of allowing blood sampling immediately after intestinal absorption, avoiding dilution in the systemic circulation and further metabolic changes of the absorbed phytochemicals. Therefore, we used portal vein cannulated rats to evaluate the intestinal bioavailability and absorption kinetics after an oral dose and jugular cannulated rats to evaluate the systemic metabolism and plasma levels of the same compounds after an intravenous dose. In both cases the rats were moving freely and unanesthetized during the experiment.

The biokinetics of olive polyphenols, oleuropein [23–27], and hydroxytyrosol [24, 28–31] has been investigated in rats. However, the biokinetics of oleuropein aglycones in living rats has never been studied in spite of their significant biological activities [32]. As oleuropein aglycones are one of the most abundant phytochemicals in the olive fruit, monitoring its absorption, metabolism, and excretion would be indispensable for understanding physiological functions of olive fruit.

In this experiment, we monitored the oleuropein aglycone 3,4-DHPEA-EDA and related polyphenols in the portal plasma and urine after oral administration, and in the systemic venous plasma and urine after intravenous administration; both procedures were done in freely moving unanesthetized rats. In addition, biliary excretion was also monitored in bile cannulated rats after intravenous administration of the phytochemicals. Together, those three procedures enabled a comprehensive view of the bioavailability and biokinetics of 3,4-DHPEA-EDA, including intestinal absorption and first-pass metabolism, systemic metabolism, and urinary and biliary excretion. This approach provides a more accurate indication of the biologically important compounds from olive fruits.

## 2. Materials and Methods

### 2.1. Chemicals

Oleuropein (Ole) was obtained from Nacalai Tesque, Inc. (Kyoto, Japan). Hydroxytyrosol (HT, 2-(3,4-dihydroxyphenyl) ethyl alcohol) was purchased from Tokyo Chemical Co. (Tokyo, Japan). Oleuropein aglycone (3,4-DHPEA-EDA, 3,4-dihydroxyphenylethyl elenolate dialdehydic form) was purified using ODS-preparative HPLC according to a previous study [32]. Homovanillic acid (HVA, 4-hydroxy-3-methoxyphenylacetic acid), homovanillyl alcohol (HVAOH, 4-hydroxy-3-methoxyphenethyl alcohol), MOPS [3-(*N*-morpholino)propanesulfonic acid], *β*-glucuronidase Type VII-A, and sulfatase Type H-1 were obtained from Sigma-Aldrich (St. Louis, MO, USA). Trifluoroacetic acid (TFA) was obtained from Wako Pure Chemical Co. (Tokyo, Japan).

### 2.2. Animals and Diets

SPF Wistar ST rats (6 weeks of age, male, 180 ± 20 g of body weight) were purchased from Japan SLC Inc. (Hamamatsu, Japan). The rats were housed in an air-conditioned room at 22 ± 2°C under cycles of 12 hours dark and 12 hours light and given free access to a commercial diet and water for a week. The rats were then fasted for over 16 hours before the experiments. The present study was approved by the Animal Care and Use Committee at Kagawa University, and the rat treatment conformed to the Rules of Animal Experiments at Kagawa University.

### 2.3. Animal Treatment: Jugular Vein, Portal Vein, and Bile Duct Cannulation

After fasting, the rats were cannulated with a polyethylene tube (PE-50; 0.58 mm I.D., 0.965 mm O.D.) into the jugular vein, portal vein, or bile duct under anesthesia with isoflurane inhalation using a WP-SAA01 (LMS Co., Ltd., Tokyo, Japan). A small hole was made in the vein or duct using pointed scissors and cannulated with the polyethylene tube. After inserting the tube, it was fixed at the edge of the vein or duct. The jugular vein and portal vein cannulas penetrated under the skin and were then tied at the back of the rats, and the bile duct cannula was tied at the abdomen of the rats. Bile duct-cannulated rats were then restrained in a Bollman cage (Natsume Seisakusho Co., Ltd., Tokyo, Japan). Before further experiments, the rats were allowed to recover from anesthesia.

### 2.4. Identification of Metabolites

Metabolites from 3,4-DHPEA-EDA, HT, and Ole were monitored using a UPLC/Xevo Q-TOF MS (Waters Corp., Milford, MA, USA). Retention time and UV *λ*_max_ from a diode array detector UFLC, and molecular ions of Xevo Q-TOF MS of the metabolites were compared with those of authentic compounds (Table 1).

### 2.5. Collection of Blood, Urine, and Bile Samples

3,4-DHPEA-EDA (300 mg/kg body weight; 936.51 *μ*mol/kg) dissolved in 50% polyethylene glycol aqueous solution was administered orally via a stomach tube to the portal vein cannulated rats. Blood samples (450 *μ*L) were then collected using a heparinized syringe attached to the cannula before administration and 5, 15, 30, 60, 120, and 240 min after administration. Urine was also collected before administration and 0 to 2 h and 2 to 4 h after administration. For intravenous administration, 3,4-DHPEA-EDA (10 mg/kg; 31.22 *μ*mol/kg) was injected into the jugular vein, and blood (450 *μ*L) was collected using a heparinized syringe before administration and 5, 15, 30, 60, 120, and 240 min after administration via the jugular vein cannula. The blood (450 *μ*L) collected was immediately cooled on ice and centrifuged at 15,000 rpm for 5 min at 4°C to obtain over 200 *μ*L plasma. Urine was also collected before administration and 0 to 2 h and 2 to 4 h after administration. Bile was collected via the bile duct cannula before administration and 0 to 2 h and 2 to 4 h after intravenous administration. The collected plasma, urine, and bile were immediately stored in an ice box until further analyses.

HT (oral administration: 100 mg/kg; 648.66 *μ*mol/kg, intravenous administration: 10 mg/kg; 64.87 *μ*mol/kg) and Ole (oral administration: 300 mg/kg; 555.02 *μ*mol/kg, intravenous administration: 10 mg/kg; 18.50 *μ*mol/kg) were investigated using the same method as described above.

All animal studies were performed in triplicate per compound.

### 2.6. Determination of 3,4-DHPEA-EDA, HT, and Ole and Their Metabolites in Plasma, Urine, and Bile

Metabolites were deconjugated by using *β*-glucuronidase (500 U) and sulfatase (50 U) in 50 *μ*L 625 mM MOPS (pH 6.8), which was added to 200 *μ*L of plasma, urine, or bile. The mixture was incubated for 45 min at 37°C and subjected to extraction using QuEChERS [33]. After that, each sample was evaporated to dryness* in vacuo*, dissolved in 200 *μ*L 0.5% TFA-1% acetonitrile aqueous solution, and then analyzed using a UFLC system (Prominence UFLC; Shimadzu, Kyoto, Japan) equipped with a Shim-pack XR-ODS column (3.0 mm I.D. × 100 mm; Shimadzu, Kyoto, Japan) at 40°C with a mobile phase of 0.5% TFA-1% acetonitrile aqueous solution (A) and 0.5% TFA-75% acetonitrile aqueous solution (B). The elution conditions are described as follows: isocratic elution of 0% B was maintained for 4.0 min; linear gradient 0 to 4% B, 4.0–4.5 min; linear gradient 4.0–16.0% B, 4.5–16.0 min; linear gradient 16.0–100% B, 16.0–25.0 min; isocratic elution of 100% B, 25.0–26.0 min; and linear gradient 100 to 0% B, 26.0–26.1 min, with a flow rate of 0.5 mL/min. Injection volume was 50 *μ*L. All eluted peaks were detected at 279 nm with a diode array detector (SPD-M20A; Shimadzu, Kyoto, Japan).

### 2.7. Calculation of Pharmacokinetic Parameters after Administration

The absorption of 3,4-DHPEA-EDA, HT, and Ole was evaluated by dose-normalized 4 h area under the curve (AUC_0→4 h_/Dose) and *C*_max_/Dose values (Table 2). Dose_i.g._ and Dose_i.v._ are the amount of oral (i.g.) and intravenous (i.v.) administration, respectively. *C*_max_ is the maximum concentration, and *T*_max_ is the time at which *C*_max_ is observed. AUC (*μ*M·min) is the area under the plasma concentration curve.

## 3. Results

### 3.1. Absorption and Metabolism of 3,4-DHPEA-EDA, HT, and Ole after Oral Administration

**Figure 1:**
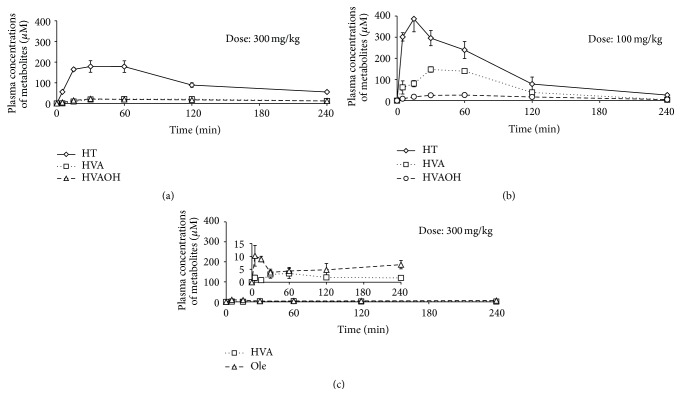
Metabolites from 3,4-DHPEA-EDA (a), hydroxytyrosol (b), and oleuropein (c) in plasma from the portal vein over time after oral administration (300 mg/kg for 3,4-DHPEA-EDA, 100 mg/kg for HT, and 300 mg/kg for Ole). Values of means and SEM are from measurements performed in triplicate. Sampling times are described in the text. HT: hydroxytyrosol, Ole: oleuropein, HVA: homovanillic acid, and HVAOH: homovanillyl alcohol.

The metabolites HT, HVA, and HVAOH were observed when 3,4-DHPEA-EDA was administrated orally (Figure 1(a)). 3,4-DHPEA-EDA was not detected in the portal plasma after oral administration (300 mg/kg; 936.51 *μ*mol/kg), even though HT as a metabolite was detected at a fairly high concentration, with a dose-normalized *C*_max_ (*C*_max_/Dose_i.g._) of 0.22 *μ*M·kg/*μ*mol and AUC (AUC_0→4 h-i.g._/Dose_i.g._) of 27.7 *μ*M·min·kg/*μ*mol (Table 2). The other metabolites of 3,4-DHPEA-EDA were HVA (*C*_max_/Dose_i.g._ 0.03 *μ*M·kg/*μ*mol; AUC_0→4 h-i.g._/Dose_i.g._ 4.5 *μ*M·min·kg/*μ*mol) and HVAOH (*C*_max_/Dose_i.g._ 0.03 *μ*M·kg/*μ*mol; AUC_0→4 h-i.g._/Dose_i.g._ 4.2 *μ*M·min·kg/*μ*mol) (Table 2). *T*_max_ (time at maximum concentration) for HT in the portal plasma was 30 min after oral administration of 3,4-DHPEA-EDA (Figure 1(a)). HT in the portal plasma subsequently decreased gradually in concentration until 240 min. In addition, HVA and HVAOH showed *T*_max_ at 60 min and 30 min, respectively, even though those detected amounts in the portal plasma were very low. Observation of HVAOH in the portal plasma was the first from 3,4-DHPEA-EDA.

The plasma concentration of HT (Figure 1(b)) immediately increased just after HT oral administration (100 mg/kg; 648.66 *μ*mol/kg) and showed AUC_0→4 h-i.g._/Dose_i.g._ of 51.1 *μ*M·min·kg/*μ*mol (Table 2). The AUC_0→4 h-i.g._/Dose_i.g._ values for HVA and HVAOH were 22.8 and 7.1 *μ*M·min·kg/*μ*mol, respectively (Table 2). Moreover, the *C*_max_/Dose_i.g._ of HT was 0.61 *μ*M·kg/*μ*mol at *T*_max_ 15 min. Those of HVA and HVAOH were 22.8 *μ*M·kg/*μ*mol at 30 min and 7.1 *μ*M·kg/*μ*mol at 60 min, respectively.

Small amounts of Ole (Figure 1(c)) (AUC_0→4 h-i.g._/Dose_i.g._ 1.8 *μ*M·min·kg/*μ*mol) and HVA (AUC_0→4 h-i.g._/Dose_i.g._ 0.9 *μ*M·min·kg/*μ*mol) were detected after administration of Ole (300 mg/kg; 555.02 *μ*mol/kg). Ole *T*_max_ was observed at 5 min. However, HVA peaked at 60 min.

### 3.2. Plasma Concentration of 3,4-DHPEA-EDA, HT, and Ole after Intravenous Administration

**Figure 2:**
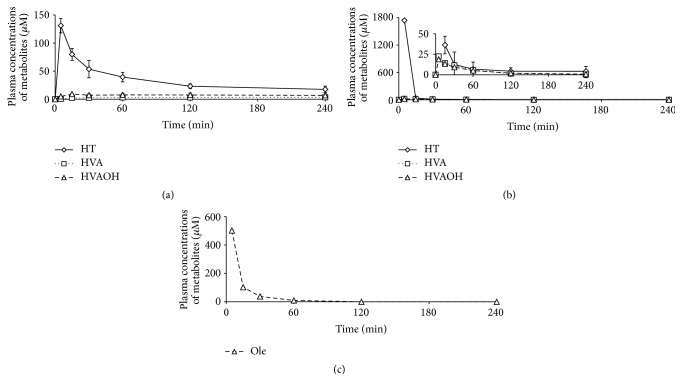
Metabolites from 3,4-DHPEA-EDA (a), hydroxytyrosol (b), and oleuropein (c) in plasma from the jugular vein over time after intravenous administration. Values of means and SEM are from measurements performed in triplicate. Analytical times are described in the text. HT: hydroxytyrosol, Ole: oleuropein, HVA: homovanillic acid, and HVAOH: homovanillyl alcohol.

The intact form of 3,4-DHPEA-EDA was not detected in the plasma and hematocytes after intravenous administration of 3,4-DHPEA-EDA (10 mg/kg; 31.22 *μ*mol/kg) (Figure 2(a)). On the other hand, about 131 *μ*M *C*_max_ of HT was detected at 5 min after intravenous administration of 3,4-DHPEA-EDA. HT was gradually metabolized in the systemic circulation, because small amounts of HVA and HVAOH could be detected. The AUC_0→4 h-i.v._/Dose_i.v._ values for HT, HVA, and HVAOH were 259.2, 22.4, and 56.0 *μ*M·min·kg/*μ*mol, respectively. The higher amount of HVAOH observed was a typical characteristic of 3,4-DHPEA-EDA metabolism in plasma.

A maximum amount of intact HT was detected at 5 min (1744 *μ*M) after intravenous administration of HT (10 mg/kg; 64.87 *μ*mol/kg), which immediately decreased in the systemic venous plasma, and then almost disappeared about 15 min after dose (Figure 2(b)). The AUC_0→4 h-i.v._/Dose_i.v._ values for HVA and HVAOH were 15.4 and 15.0 *μ*M·min·kg/*μ*mol, respectively.

After administration of Ole (10 mg/kg; 18.50 *μ*mol/kg) (Figure 2(c)), 502 *μ*M Ole was detected in the plasma at 5 min and then disappeared from the systemic circulation within 60 min. Other metabolites could not be detected throughout the blood sampling.

### 3.3. Urinary Excretion of 3,4-DHPEA-EDA, HT, and Ole after Oral Administration

**Figure 3:**
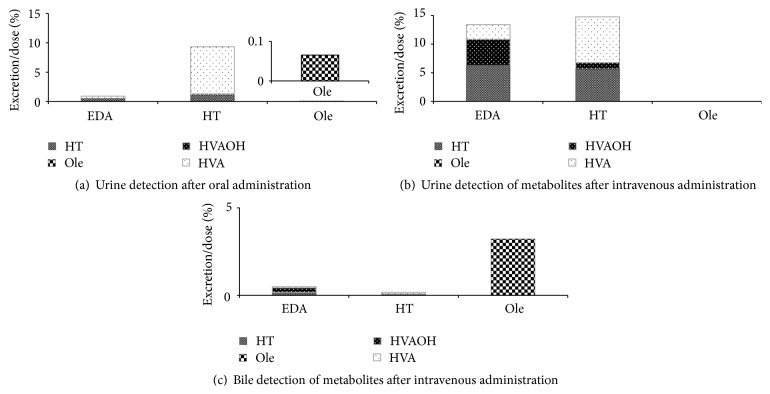
Monitoring of metabolites in urine and bile after oral and intravenous administration of 3,4-DHPEA-EDA, hydroxytyrosol, and oleuropein. Excretion rates are expressed as excretion/dose ratios. EDA: 3,4-DHPEA-EDA, HT: hydroxytyrosol, Ole: oleuropein, HVA: homovanillic acid, and HVAOH: homovanillyl alcohol.

After oral administration of 3,4-DHPEA-EDA, HT (0.51% of dose) and HVA (0.38% of dose) were detected in small amounts in the urine (Figure 3(a)), whereas 3,4-DHPEA-EDA and HVAOH were not detected in the urine.

HVAOH (0.17% of dose) and HVA (8.03% of dose) were the major metabolites of intact HT (1.14% of dose) in the urine of the rats after oral administration of HT (Figure 3(a)). Ole was detected in an extremely small amount (0.07% of dose) in the urine (Figure 3(a)) after oral administration of Ole.

### 3.4. Urinary Excretion of 3,4-DHPEA-EDA, HT, and Ole after Intravenous Administration

The intact form of 3,4-DHPEA-EDA was not detected in the urine (Figure 3(b)). On the other hand, 6.3% HT, 2.6% HVA, and 4.6% HVAOH against the dose were observed in the urine after intravenous administration. A fairly large amount of HVAOH detected is a characteristic of urinary excretion of 3,4-DHPEA-EDA after intravenous administration.

HT converted quickly into HVA (8.0%) in the urine after intravenous administration (Figure 3(b)), and then the transformation of HT into HVAOH after intravenous administration was 1.1% in the urine (Figure 3(b)). HT administrated intravenously leads to detecting a fair amount of intact HT.

The intact form of Ole and the metabolites were not detected in the urine.

### 3.5. Biliary Excretion of 3,4-DHPEA-EDA, HT, and Ole after Intravenous Administration

With the 3,4-DHPEA-EDA substrate, 0.174% HT, 0.048% HVA, and 0.255% HVAOH were detected in the bile (Figure 3(c)).

In contrast, 0.079% HT, 0.090% HVA, and 0.003% HVAOH were detected after HT was metabolized (Figure 3(c)). The chemical compositions of HT, HVA, and HVAOH in the bile from EDA and HT after intravenous administration were quite similar to those in the urine.

With the Ole substrate, the intact form of Ole was not detected in the urine, but 3.22% of the injected dose was observed in the bile (Figure 3(c)).

## 4. Discussion

### 4.1. Absorption of 3,4-DHPEA-EDA and Its Related Polyphenols

In comparing 3,4-DHPEA-EDA, HT, and Ole, the highest absorption was observed for HT, followed by 3,4-HPEA-EDA and Ole (Figure 1). Because the dose of 3,4-DHPEA-EDA (936.5 *μ*mol/kg) was 1.44 times greater than that of HT (648.7 *μ*mol/kg), the total absorption rate of 3,4-DHPEA-EDA (36.4 AUC_0→4 h-i.g._/Dose_i.g._), including its metabolites, was only 2.2 times less than that of HT (81.0 AUC_0→4 h-i.g._/Dose_i.g._) (Table 2). The difference in mutual absorption rates between 3,4-DHPEA-EDA and HT is quite small. On the other hand, the absorption rate of 3,4-DHPEA-EDA was 13.5 times greater than that of Ole (2.7 AUC_0→4 h-i.g._/Dose_i.g._). Therefore, total absorption of 3,4-DHPEA-EDA would be significant for the physiological function of olive fruit (ratio of AUC/Dose of 3,4-DHPEA-EDA : HT : Ole = 13.5 : 30 : 1). In addition, there is high specificity in the absorption rate among them.

After oral administration of 3,4-DHPEA-EDA, HT (27.7 AUC_0→4 h-i.g._/Dose_i.g._), HVAOH (4.2 AUC_0→4 h-i.g._/Dose_i.g._), and HVA (4.5 AUC_0→4 h-i.g._/Dose_i.g._) were detected in the portal plasma. Low absorption of 3,4-DHPEA-EDA is reported in some papers [16, 34]. However, we could not detect any 3,4-DHPEA-EDA or 3,4-DHPEA-EDA-related compounds in a bound form, or an unbound form like 3,4-DHPEA-EDAH_2_ [34], in the portal plasma. Plasma components such as serum albumin, serum lipoprotein, and glycoprotein may have bound 3,4-DHPEA-EDA and hindered its detection. 3,4-DHPEA-EDA as a dialdehyde form might quickly bind to proteins bearing primary amines such as globulins, albumins, and other functional proteins in blood plasma, as malondialdehyde does [35, 36]. We spiked 3,4-DHPEA-EDA into rat plasma to check the recovery yield, but 3,4-DHPEA-EDA could not be detected. To our knowledge, this is the first report of the formation of HVAOH from 3,4-DHPEA-EDA.

HT (51.1 AUC_0→4 h-i.g._/Dose_i.g._), HVAOH (7.1 AUC_0→4 h-i.g._/Dose_i.g._), and HVA (22.8 AUC_0→4 h-i.g._/Dose_i.g._) were observed in the portal plasma (Figure 1(b)) when HT was administrated orally. Detecting HVA and HVAOH in plasma after ingestion of olive oil and HT has been documented in some papers [14, 15], but detection of them has not been reported so far from 3,4-DHPEA-EDA. Although a great amount of HVA was detected from HT in the portal plasma, a small amount of HVA was detected from 3,4-DHPEA-EDA (Figure 1(a)). If an intermediate metabolite from orally administrated 3,4-DHPEA-EDA is HT, HVA should be observed in the portal plasma. Our findings probably indicated that 3,4-DHPEA-EDA or 3,4-DHPEA-EDA-related compounds in a bound form, or an unbound form like 3,4-DHPEA-EDAH_2_ [34], would be gradually absorbed in the portal plasma.

The absorption of 3,4-DHPEA-EDA can be further supported by the metabolism of 3,4-DHPEA-EDA, HT, and Ole. Accordingly, 3,4-DHPEA-EDA was not observed in the plasma from the jugular vein after intravenous administration (Figure 2(a)). In addition, HT (259.2 AUC_0→4 h-i.g._/Dose_i.g._), HVAOH (56.0 AUC_0→4 h-i.g._/Dose_i.g._), and HVA (22.4 AUC_0→4 h-i.g._/Dose_i.g._) from 3,4-DHPEA-EDA were monitored in the plasma from the jugular vein after intravenous administration throughout the HPLC monitoring (240 min) (Figure 2(a)). Detection of high levels of HVAOH from 3,4-DHPEA-EDA was unique and it differs from the detection of HT, HVAOH, and HVA from HT (Figures 3(a) and 3(b)). Another interesting feature is the slower clearance rate of HT as a 3,4-DHPEA-EDA metabolite (Figure 2(a)) in the systemic circulation, compared with that of intact HT (Figure 2(b)). If orally administrated 3,4-DHPEA-EDA can be converted to HT in the gastrointestinal tract, HT from 3,4-DHPEA-EDA and the intact HT should have rapid and similar clearance rates in the systemic venous plasma. These results might suggest that 3,4-DHPEA-EDA is present in the systemic venous blood (e.g., bound to plasma proteins) or some organs as the bound form, which would be gradually released. The slow release of 3,4-DHPEA-EDA and its related polyphenols to the free form and formation of the metabolites (HT, HVAOH, and HVA) maintain the biological function for a long period in the body. The formation of homovanillyl alcohol from 3,4-DHPEA-EDA administration might influence function, for example, as a neurotransmitter [37], which is also supporting evidence for the absorption of 3,4-DHPEA-EDA by rats.

### 4.2. Excretion of 3,4-DHPEA-EDA and Its Related Polyphenols after Oral and Intravenous Administration

After oral administration of 3,4-DHPEA-EDA, HT (0.51% of dose) and HVA (0.39% of dose) were detected in small amounts in the urine of the rats (Figure 3(a)), while HVAOH was not detected in the urine (Figure 3(a)). In total, 0.90% of the 3,4-DHPEA-EDA metabolites was excreted in the urine within 240 min after oral administration. Conversely, observation of the metabolites HT, HVAOH, and HVA from 3,4-DHPEA-EDA in the portal plasma revealed that HT (27.7 AUC_0→4 h-i.g._/Dose_i.g._), HVAOH (4.2 AUC_0→4 h-i.g._/Dose_i.g._), and HVA (4.5 AUC_0→4 h-i.g._/Dose_i.g._) were detected after oral administration (Figure 1(a)). These results indicate that 3,4-DHPEA-EDA lasts a long time in the systemic venous blood (e.g., bound to plasma proteins) or some organs as the bound form, which suggests that the physiological functions of 3,4-DHPEA-EDA, including the metabolites, will continue for a long period in the body.

On the other hand, after oral administration of HT, a large amount of HT (1.14% of dose), HVAOH (0.17% of dose), and HVA (8.03% of dose) were detected in the urine. In total, 9.34% of the HT metabolites including intact HT was rapidly excreted into the urine within 240 min after administration. The total excretion ratio of 3,4-DHPEA-EDA, HT, and Ole including their metabolites was 12.9 : 133.4 : 1 (3,4-DHPEA-EDA : HT : Ole). Comparing with the ratio of AUC/Dose of 3,4-DHPEA-EDA : HT : Ole (ratio of AUC/Dose of 3,4-DHPEA-EDA : HT : Ole = 13.5 : 30 : 1) in the portal plasma (Figure 1), it is clear that 3,4-DHPEA-EDA (bound form, its related compounds, and its metabolites), HT, and the metabolites in the body have high potential for contributing to physiological function as mentioned in Section 1.

With respect to 3,4-DHPEA-EDA, HT, and Ole excretion to urine and bile, monitoring and tracing these three chemicals and their metabolites after intravenous administration is simpler and easier than monitoring and tracing them after oral administration. Moreover, the first-pass hepatic metabolites after intravenous administration can be easily monitored and interpreted compared with metabolites detected in the systemic venous blood after oral administration. Therefore, oral administration results in more complicated and fewer metabolites than intravenous administration. Accordingly, a limited amount of chemicals could be detected when they were observed in the urine and bile because the absorption phase is subject to certain restrictions for selective transportation, conjugation, modification, and metabolism of the chemicals. Therefore, careful interpretation of the results is required.

Consequently, when 3,4-DHPEA-EDA was directly administrated to the jugular vein, 3,4-DHPEA-EDA was not observed, but HT (6.3% of dose), HVAOH (4.6% of dose), and HVA (2.6% of dose) were observed in the urine as excreted products (Figure 3(b)). The same chemicals, HT (0.17% of dose), HVAOH (0.26% of dose), and HVA (0.05% of dose), were observed in small amounts in the bile (Figure 3(c)). It can be concluded from the data above that mainly 3,4-DHPEA-EDA and its metabolites were excreted to the urine. This tendency was similarly observed when HT was directly administered to the jugular vein (Figures 3(b) and 3(c)). Thus, HT and its metabolites described above are mainly excreted to the urine. Conversely, Ole was not detected at all in the urine when Ole was administrated to the jugular vein. Instead, Ole (3.22% of dose) was excreted in the bile without any other metabolites (Figure 3(c)). Finally, excretion of Ole to the bile would be the most predominant pathway of Ole without any transformation of the chemical structure. The metabolic pathway of Ole is regulated and suppressed by inactivation of deglycosylation, hydrolysis, oxygenation, and methylation enzymes, which are related to metabolism after intravenous administration [26].

3,4-DHPEA-EDA, HT, and Ole could be easily excreted to urine or bile when these chemicals were intravenously administrated. When 3,4-DHPEA-EDA and HT in the urine after oral administration and intravenous administration were compared, 3,4-DHPEA-EDA showed a great difference in detection ratios of the metabolites against doses between i.g. and i.v. (Figures 3(a) and 3(b)). Thus, 13% metabolites are observed in the urine after intravenous administration but only 0.9% metabolites are observed in the urine after oral administration. HT had a similar tendency in the detection ratios against each dose. It is supposed that the ratios of metabolites from 3,4-DHPEA-EDA and HT in the urine after oral administration (Figure 3(a)) reflect those of 3,4-DHPEA-EDA, including its related substances, and HT absorbed in the body (Figures 2(a) and 2(b)) but do not reflect the metabolite ratios of 3,4-DHPEA-EDA and HT administered in the jugular vein, which means 3,4-DHPEA-EDA in the jugular plasma after oral administration is not the same as 3,4-DHPEA-EDA itself; it is modified like the bound form to certain proteins in the vein. Furthermore, 3,4-DHPEA-EDA or its related compounds with or without bound forms were retained in the body when 3,4-DHPEA-EDA was orally administrated, but HT was released easily to the urine as the excreted substance after oral administration in rats as reported in a previous paper [14]. In total, the physiological function of 3,4-DHPEA-EDA, its related polyphenols, and its metabolites last as active substances in the body.

### 4.3. Proposed Metabolic Pathway of 3,4-DHPEA-EDA

With the orally administrated 3,4-DHPEA-EDA, the late appearance of HVA (*T*_max_ = 60 min), compared with HT (*T*_max_ = 30 min) and HVAOH (*T*_max_ = 30 min), supports the stepwise metabolism of 3,4-DHPEA-EDA, including 3,4-DHPEA-EDA-related polyphenols (bound form or unbound form), to HT, HVAOH, and HVA in this order. Furthermore, the ratios of the metabolites (HT, HVAOH, and HVA) from 3,4-DHPEA-EDA and HT were mainly influenced by enzyme activities in the gastrointestinal tract and the systemic venous blood. With oral administration of 3,4-DHPEA-EDA and HT, different ratios of metabolites were detected in the portal plasma. Thus, HT was observed as the main metabolite after oral administration of 3,4-DHPEA-EDA, but a large amount of HVA was observed as one of major metabolites of HT as expected from a previous paper [21] (Figures 2(a) and 2(b)). Enzymatic methylation and oxidation of HT by using catechol 3-*O*-methyltransferase (COMT), alcohol dehydrogenase, and aldehyde dehydrogenase have been reported in the gastrointestinal tract as the metabolization of HT using caco-2 cells [38]. However, 3,4-DHPEA-EDA metabolism was not clarified in previous studies. In fact, 3,4-DHPEA-EDA did not easily hydrolyze to HT in the gastrointestinal tract because HVA detection in the portal plasma from 3,4-DHPEA-EDA was not so high, when compared with HT metabolism under the same conditions.

**Figure 4:**
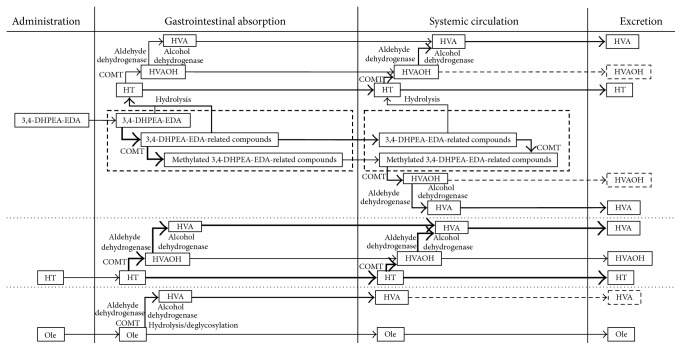
The possible flow of chemical conversion of 3,4-DHPEA-EDA, hydroxytyrosol, and oleuropein after administration in rats. HT: hydroxytyrosol, HVA: homovanillic acid, and HVAOH: homovanillyl alcohol.

Another point of view regarding 3,4-DHPEA-EDA metabolism is related to the large amount of HVAOH (4.6% of dose) from 3,4-DHPEA-EDA in the urine after intravenous administration, compared with that from HT. Systemic venous blood and the kidneys may easily metabolize 3,4-DHPEA-EDA or 3,4-DHPEA-EDA-related polyphenols (bound form or unbound form) to HVAOH but not to HVA (Figure 3(b)). The same tendency of HVAOH formation from 3,4-DHPEA-EDA and its related polyphenols was observed when it was detected in the jugular plasma after intravenous administration with a long period of detection of HT (Figure 2(a)). In contrast, HT was easily metabolized to HVA in the urine after intravenous administration (Figure 3(b)). Systemic venous blood and the kidneys may have different active enzymes for digesting 3,4-DHPEA-EDA, including 3,4-DHPEA-EDA-related polyphenols and HT. HVAOH formation from 3,4-DHPEA-EDA is much easier in the systemic venous blood than that of HT. COMT and aldehyde-alcohol dehydrogenase activities against 3,4-DHPEA-EDA and HT would be different in affinity. In considering the metabolism of 3,4-DHPEA-EDA and its related polyphenols from the data shown in the figures and tables, we have summarized the conversion of three typical substances in olive fruit (Figure 4).

## 5. Conclusion

The biokinetics of absorption, metabolism, and excretion of three olive phytochemicals (3,4-DHPEA-EDA, HT, and Ole) were studied in freely moving rats cannulated into the portal vein and jugular vein and restrained bile duct-cannulated rats. The ratio of AUC/Dose of 3,4-DHPEA-EDA : HT : Ole was 13.5 : 30 : 1 and these values are related to the rates of absorption of each substance. Furthermore, the total excretion ratio of 3,4-DHPEA-EDA, HT, and Ole including their metabolites was 12.9 : 133.4 : 1, which means that 3,4-DHPEA-EDA, including the metabolites, should be the most important source of biologically active substances in olive fruit overall when considering efficiency in the body. The amount of 3,4-DHPEA-EDA in olive fruit is also reported to be the highest [32].

In the circulation of metabolites in the blood, we found different enzymatic modification of 3,4-DHPEA-EDA and HT: 3,4-DHPEA-EDA mainly transforms to HVAOH, while HT transforms to HVA after intravenous administration. Conversely, transformation of Ole to other metabolites did not occur in the systemic venous blood. The low detection of Ole in the portal plasma after oral administration was due to low intestinal absorption and not fast metabolization by plasma enzymes. It has been supported in some papers [23, 26]. Finally, only minute amounts of Ole could be absorbed from the gastrointestinal tract after oral administration, and it was extensively excreted via the biliary route when administered intravenously, rendering as questionable its importance as a bioactive compound from olives.

In conclusion, the physiological function of 3,4-DHPEA-EDA and its related polyphenols and metabolites should make it an important functional food component in olive fruit.

## Figures and Tables

**Table 1:** Identification of metabolites from plasma, urine, and bile after administration of 3,4-DHPEA-EDA, hydroxytyrosol, and oleuropein.

Rt (min)	*λ*_max_ (nm)	Formula	Calc. mass [M−H]^−^	Observed [M−H]^−^	Mass error (mDa)	Compound identification
3.96	230, 278	C_8_H_10_O_3_	153.0552	153.0554	0.2	Hydroxytyrosol
8.95	228, 278	C_8_H_12_O_3_	167.0708	167.0731	2.3	Homovanillyl alcohol
9.67	230, 278	C_9_H_10_O_4_	181.0501	181.0533	3.2	Homovanillic acid
20.10	230, 280	C_25_H_32_O_8_	539.1765	539.1776	1.1	Oleuropein

Data was obtained from HT metabolites isolated from urine.

Data was obtained from oleuropein isolated from plasma.

**Table 2:** Pharmacokinetic parameters after oral administration of 3,4-DHPEA-EDA, hydroxytyrosol, and oleuropein.

Oral administration	Dose (*μ*mol)	Metabolites	AUC (*μ*M·min)	AUC/Dose (*μ*M·min·kg/*μ*mol)	*C*_max⁡_ (*μ*M)	*C*_max⁡_/Dose (*μ*M·kg/*μ*mol)	*T*_max⁡_ (min)
3,4-DHPEA-EDA	936.51	HT	25935.0 ± 3103.1	27.7 ± 3.3	178.87 ± 45.3	0.22 ± 0.05	30
		HVA	4180.0 ± 1646.5	4.5 ± 1.8	21.5 ± 6.9	0.03 ± 0.007	60
		HVAOH	3934.5 ± 789.7	4.2 ± 0.8	22.2 ± 2.0	0.03 ± 0.002	30
		Total	**34049.5 ± 5539.3**	**36.4 ± 5.9**			

Hydroxytyrosol	648.66	HT	33160 ± 5541.5	51.1 ± 8.5	394.7 ± 58.0	0.61 ± 0.089	15
		HVA	14794.1 ± 510.1	22.8 ± 0.8	150.6 ± 9.7	0.23 ± 0.015	30
		HVAOH	4604.4 ± 885.2	7.1 ± 1.4	26.6 ± 5.2	0.04 ± 0.008	60
		Total	**52585 ± 6936.8**	**81.0 ± 10.7**			

Oleuropein	555.02	Ole	1015.8 ± 222.6	1.8 ± 0.4	11.9 ± 3.2	0.021 ± 0.006	5
		HVA	515.7 ± 112.4	0.9 ± 0.2	4.1 ± 1.4	0.007 ± 0.003	60
		Total	**1531.5 ± 335**	**2.7 ± 0.6**			

Values of means and ± SEM are in triplicate.
